# A Machine Learning Framework for Predicting Functional Status and Estimating Health Care Costs in Pulmonary Arterial Hypertension: Retrospective Cohort Study

**DOI:** 10.2196/97372

**Published:** 2026-10-15

**Authors:** Mira Kramer, Tilmann Kramer

**Affiliations:** 1 Department of Anesthesiology and Intensive Care Medicine University Hospital Ulm Ulm, Baden-Württemberg Germany; 2 Department of Internal Medicine III, Cardiology Heart Center University Hospital Cologne Cologne, North Rhine-Westphalia Germany; 3 Department of Internal Medicine II Cardiology University Hospital Ulm Ulm, Baden-Württemberg Germany

**Keywords:** pulmonary arterial hypertension, machine learning, health care costs, cost prediction, WHO-FC, health economics, World Health Organization functional class

## Abstract

**Background:**

Pulmonary arterial hypertension (PAH) is a rare and progressive disease associated with substantial clinical heterogeneity and high health care costs. While machine learning (ML) has been increasingly applied to predict clinical outcomes, approaches that integrate clinical prediction with downstream economic modeling remain limited.

**Objective:**

This study proposes and evaluates an ML-based framework that combines prediction of future functional status with estimation of associated health care costs in patients with PAH.

**Methods:**

In this retrospective cohort study, data from 181 patients with PAH were analyzed. A total of 6 supervised ML algorithms (Least Absolute Shrinkage and Selection Operator [LASSO] regression, ridge regression, k-nearest neighbors, decision trees, random forest, and gradient boosting) were trained to predict the relative change in World Health Organization functional class (WHO-FC) from baseline to 12-month follow-up using baseline clinical variables. Predicted change rates were subsequently converted to predicted WHO-FC values at the 12-month follow-up for downstream health care cost estimation. Missing values in predictor variables were imputed using median substitution. Median imputation was performed before the dataset was split into training and test sets. Model performance was evaluated using Pearson correlation coefficient (*r*), and error metrics (RMSE, MAE, and MedAE), based on cross-validation and independent test set validation. Predicted WHO-FC values were subsequently linked to externally validated, stratified real-world health care cost estimates to approximate future economic burden.

**Results:**

Among the evaluated models, k-nearest neighbors showed the highest predictive performance (*r*=0.40; *R*^2^=0.16), followed by LASSO regression (*r*=0.30; *R*^2^=0.09). Prediction errors were comparable across models. Estimated mean health care costs ranged from US $12,481 to $12,614 per patient per month. Despite a significant improvement in WHO-FC over 12 months, estimated health care costs remained largely unchanged, reflecting persistent economic burden across functional class strata.

**Conclusions:**

This study demonstrates the feasibility of an ML-based framework that integrates prediction of functional status with health care cost estimation in PAH. While predictive performance was moderate, the approach illustrates how ML models can be extended beyond clinical end points toward system-level applications such as cost forecasting and resource planning. Further validation in larger, multicenter cohorts is required before clinical or health system implementation.

## Introduction

### Clinical and Economic Burden of Pulmonary Arterial Hypertension

Pulmonary arterial hypertension (PAH) is a rare but severe cardiopulmonary disease that ultimately leads to right heart failure and premature death [1]. As a form of pulmonary vascular disease, PAH is characterized by progressive vascular remodeling, increasing pulmonary vascular resistance (PVR), and right ventricular overload. According to the 2022 guidelines of the European Society of Cardiology (ESC) and the European Respiratory Society (ERS) [1], PAH is hemodynamically defined by a mean pulmonary arterial pressure (mPAP) greater than 20 mm Hg, PVR greater than 2 Wood units (WU), and pulmonary arterial wedge pressure (PAWP) of 15 mm Hg or less, confirmed by right heart catheterization (RHC). Despite therapeutic advances [1-3], PAH continues to impose a substantial clinical and economic burden, including high health care resource usage (HCRU) [1,4-9].

Even with a low prevalence of 0.03 to 0.05 per 1,000 population [6], PAH incurs disproportionately high health care costs. In the United States, the annual national burden of treated PAH patients to payers was estimated at US $3.1 billion [8]. Such cost patterns are typical for orphan diseases requiring complex therapies and resource-intensive medical care [10]. Recent real-world claims analyses reported mean direct health care costs exceeding US $15,000 per patient per month (PPPM) in patients with advanced stages of disease, corresponding to the World Health Organization functional class (WHO-FC) IV or equivalent disease severity [8,11], adjusted to 2024 using the Medical Care component of the US Consumer Price Index (CPI) [12,13]. Key cost drivers include pharmacotherapy, hospitalizations, specialist care, diagnostic workups, and emergency services [8,11,14].

Beyond direct medical expenditures, PAH is also associated with substantial indirect and non–health care costs, including productivity losses, disability, and time spent on medical visits, resulting in a considerable societal burden [9,15].

Despite this burden, stratified and longitudinal cost data in PAH remain limited, and many existing studies report population-level estimates without sufficient differentiation by clinical severity, follow-up time, indirect costs, or pulmonary hypertension (PH) subtype [4-6,16]. This distinction is particularly important because patients with PAH often require effective but costly targeted therapies that are not indicated for other forms of PH [1,17,18]. Consequently, the economic impact of individual disease progression and treatment decisions remains incompletely understood [16]. This lack of predictive and personalized cost data may limit value-based pricing, budget planning, and efficient health care resource allocation, particularly in financially constrained health care systems [19,20].

### Clinical Risk Stratification and WHO-FC

Current ESC and ERS guidelines recommend a multidimensional risk stratification approach in PAH, incorporating clinical, echocardiographic, functional, hemodynamic, and biochemical indicators, in particular WHO-FC, 6-minute walk distance (6MWD), and N-terminal pro-B-type natriuretic peptide (NT-proBNP), to guide treatment decisions, with the overarching goal of achieving and maintaining a low-risk profile associated with optimal survival [1]. WHO-FC is a widely available prognostic marker in PAH and has consistently been associated with health care costs across WHO-FC strata, supporting its use as a clinically meaningful proxy in health economic modeling [9,11,21,22].

However, longitudinal cost trajectories based on changes in WHO-FC remain insufficiently characterized, and it is unclear whether functional improvement directly translates into reduced health care expenditures [9,11,21,22]. Although improved outcomes under targeted therapy may reduce HCRU and indirect costs, validated tools to estimate future PAH-related expenditures from early clinical data are lacking [16,23-25].

### Machine Learning and Study Objective

Machine learning (ML) may help address this gap by using routinely collected clinical data to predict future clinical status and link these predictions to downstream economic estimates derived from WHO-FC–stratified cost data [11]. Prior studies have applied ML to clinically relevant outcomes in PAH, including prediction of future clinical worsening events [26]. In contrast, the integration of ML-based clinical prediction with health economic modeling remains largely unexplored.

To our knowledge, no previous study has evaluated an ML-based framework for estimating WHO-FC–based health care costs in PAH. Therefore, this study investigated whether baseline clinical data can be used to predict WHO-FC at 12-month follow-up and link these predictions to health care cost estimation [4-6,8,9,14,16,27].

## Methods

### Study Design and Population

This retrospective cohort study analyzed clinical data from 181 adult patients with invasively confirmed PAH, classified as Nice classification group 1. Diagnostic confirmation followed the 2015 ESC and ERS guidelines, requiring an mPAP ≥25 mm Hg, PVR greater than 3 WU, and PAWP of ≤15 mm Hg, confirmed by RHC [28].

Patients with borderline hemodynamics, defined as mPAP between 21 and 24 mm Hg and PVR between 2.1 and 3.0 WU, were excluded to maintain consistency with the diagnostic thresholds prevailing during the data collection period. Eligible patients presented in WHO-FC II, III, or IV at baseline and received standard-of-care therapy according to guideline recommendations [28]. Clinical data were collected between October 2010 and May 2022. The final cohort comprised 181 patients (117 female and 64 male). Given the exploratory nature of this proof-of-concept study, no formal sample size calculation was performed. Baseline parameters, including 92 structured, noninvasive, and invasive variables encompassing demographics, echocardiographic and hemodynamic measurements, laboratory biomarkers, blood gas analysis, functional assessments, and initial targeted PAH therapy, were collected no later than 8 weeks prior to RHC. All clinical and diagnostic predictor variables were obtained at baseline prior to therapy initiation.

### Hemodynamic Assessment

RHC was performed in all patients following a standardized protocol in accordance with current guideline recommendations [1,29,30]. Pressure measurements were obtained at end-expiration, averaging 3 cardiac cycles in sinus rhythm and 5 cycles in patients with atrial fibrillation. The pressure transducer was zeroed at the mid-thoracic level. Hemodynamic parameters assessed included systolic PAP, diastolic PAP, mPAP, PAWP, and right atrial pressure. Cardiac index (CI) was calculated from cardiac output and body surface area. Derived indices included the transpulmonary pressure gradient, calculated as mPAP minus PAWP, and the diastolic pulmonary gradient, calculated as diastolic PAP minus PAWP. Stroke volume index was determined, and PVR was calculated as (mPAP – PAWP) / cardiac output. Pulmonary artery compliance was calculated as stroke volume divided by the pulmonary pulse pressure. Mixed venous oxygen saturation was measured directly. All reported hemodynamic variables were obtained under resting conditions at end-expiration.

### Echocardiographic Assessment

Transthoracic echocardiography was performed in accordance with current guidelines [1] using a standardized protocol with the Philips iE33 system and a 2.5 MHz transducer. Echocardiographic parameters measured included right atrial area, right ventricular end-diastolic diameter, and pulmonary artery systolic pressure (PASP), which was estimated from the tricuspid regurgitation velocity using the modified Bernoulli equation combined with estimated right atrial pressure. Tricuspid annular plane systolic excursion (TAPSE) was measured to assess right ventricular systolic function. The TAPSE/PASP ratio was calculated as a noninvasive marker of right ventricular-pulmonary artery coupling [31,32]. Additionally, left ventricular end-diastolic diameter and left ventricular ejection fraction were determined to assess left heart size and function. Significant left-sided valvular disease (≥moderate) was systematically excluded by Doppler echocardiography.

### Functional and Biomarker Assessment

Submaximal exercise capacity was assessed using the 6-minute walk test, conducted according to the standardized protocol of the American Thoracic Society [33]. Patients were familiarized with the procedure prior to testing. WHO-FC was assessed by experienced physicians according to standard clinical criteria. Serum concentrations of NT-proBNP were measured using the Elecsys proBNP II immunoassay (Roche Diagnostics GmbH), applying age-adjusted reference values with a general threshold of <125 ng/L considered normal. Additional standard laboratory parameters, including markers of renal and liver function, were also obtained and included in model development. Arterialized capillary blood gas analysis was performed at baseline. Capillary samples were obtained from hyperemic earlobes after a standardized 10-minute resting period and analyzed using the ABL800 FLEX analyzer (Radiometer GmbH). Routinely measured and derived gas exchange parameters, including partial pressure of oxygen, partial pressure of carbon dioxide, capillary oxygen saturation, actual and standard bicarbonate, hemoglobin, hematocrit, standardized capillary oxygen saturation, capillary oxygen content, oxygen delivery, and the alveolocapillary oxygen partial pressure difference, were incorporated into model development.

### Risk Stratification

Risk stratification was conducted at baseline using 2 established methodologies based on current ESC and ERS guidelines [1]. The 4-stratum model categorized patients by grading WHO-FC, 6MWD, and NT-proBNP values from 1 (low risk) to 4 (high risk), distinguishing 4 risk levels: low risk, intermediate-low risk, intermediate-high risk, and high risk. The average grade across available variables was calculated and rounded to the nearest integer to determine the overall risk group [34,35].

Additionally, the French approach was applied [36]. This method incorporated 3 noninvasive criteria: WHO-FC I or II, 6MWD greater than 440 meters, and NT-proBNP below 300 ng/L. In an extended version, invasive parameters such as right atrial pressure below 8 mm Hg and CI of at least 2.5 L/min/m^2^ were also considered [36]. The resulting risk group assignments were included as input features in model development.

### Exclusion of Significant Lung Disease and Pulmonary Embolism

Significant pulmonary disease was excluded by spirometry, which required a forced vital capacity and a forced expiratory volume in 1 second of greater than 60% predicted, and by high-resolution computed tomography imaging demonstrating no relevant parenchymal abnormalities. Chronic thromboembolic pulmonary hypertension was ruled out in all patients by ventilation-perfusion scintigraphy according to guideline standards [1].

### Outcome Definition

The primary end point was WHO-FC assessed at 12 months following therapy initiation. Table 1 provides an overview of the WHO-FC system for patients with PH, ranging from Class I (no limitation of physical activity) to Class IV (symptoms at rest). The classification is widely used in both clinical and economic modeling [1,9,11,37].

**Table 1:** World Health Organization functional classification of patients with pulmonary hypertension^a^.

WHO-FC^b^	Description
WHO-FC I	No limitation of physical activity. Ordinary physical activity does not cause undue dyspnea, fatigue, chest pain, or presyncope.
WHO-FC II	Slight limitation of physical activity. No discomfort at rest, but ordinary activity causes undue dyspnea, fatigue, chest pain, or presyncope.
WHO-FC III	Marked limitation of physical activity. No discomfort at rest, but less than ordinary activity leads to undue dyspnea, fatigue, chest pain, or presyncope.
WHO-FC IV	Inability to carry out any physical activity without symptoms. Signs of right heart failure may be present. Dyspnea and/or fatigue may even occur at rest. Any physical activity increases discomfort.

^a^Adapted from the functional classification of PH based on the New York Heart Association classification, as outlined by the WHO (1998) [37], and modified according to the ESC and ERS guidelines for the diagnosis and treatment of PH (2022) [1].

^b^WHO-FC: World Health Organization functional class.

While WHO-FC is an easily assessable and well-established predictor of survival in PAH, it was selected in this study due to its validated association with health care costs and HCRU, serving as a clinically relevant proxy for economic modeling.

Predicted WHO-FC values were subsequently linked to externally validated, real-world health care cost data stratified by WHO-FC I-IV, based on the published analysis by Dufour et al [11], which provides functional class–specific PPPM costs. Mean pharmacy, medical, and total PPPM costs were reported separately for each WHO-FC stratum, with total costs representing the combined pharmacy and medical costs. Minor differences between displayed total costs and the sum of the displayed components may reflect rounding in the source publication.

All US dollar costs were originally expressed in 2014 values. To ensure comparability with current economic conditions, 2014 US dollar costs were inflation-adjusted to 2024 values using the Medical Care component of the US CPI, resulting in an adjustment factor calculated as follows:

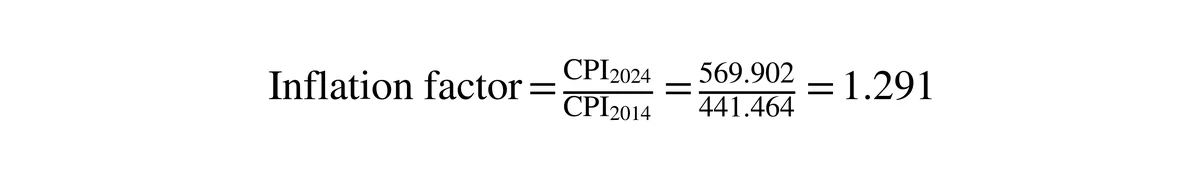



Thus, adjusted costs were calculated according to the formula:

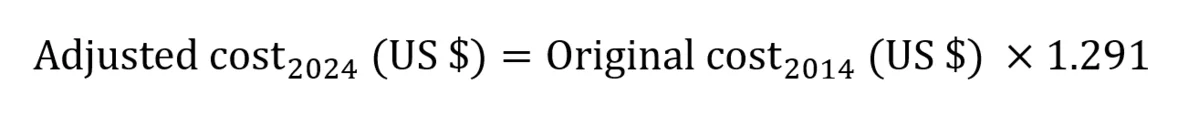



Separate inflation-adjusted estimates were obtained for pharmacy, medical, and total PPPM costs for each WHO-FC. The detailed adjusted cost values are provided in Multimedia Appendix 1. All costs are reported in US dollars.

### ML Algorithms and Model Development

ML analyses were conducted using R (version 4.3.3; R Core Team) and RStudio (version 2023.12; RStudio Team). A total of 6 supervised ML algorithms were used to predict the relative change in WHO-FC from baseline to 12-month follow-up using baseline clinical data: Least Absolute Shrinkage and Selection Operator (LASSO) regression, ridge regression (ridge), k-nearest neighbors (k-NN), decision trees, random forest (RF), and gradient boosting machine (GBM). Predicted change rates were subsequently converted to predicted WHO-FC values at the 12-month follow-up using the individual baseline WHO-FC—predicted follow-up WHO-FC = baseline WHO-FC × (1 + predicted relative change)—and rounded to the nearest whole WHO-FC value for subsequent WHO-FC–stratified health care cost estimation.

The selected algorithms were intended to represent a diverse range of commonly used supervised ML approaches, including regularized linear models (LASSO and ridge), instance-based learning (k-NN), single-tree models (decision trees), and ensemble methods (RF and GBM). This combination enabled comparison of algorithms with different assumptions regarding linearity, feature interactions, and model complexity within the constraints of the available sample size.

LASSO applied L1 regularization to perform feature selection by minimizing the following loss function:

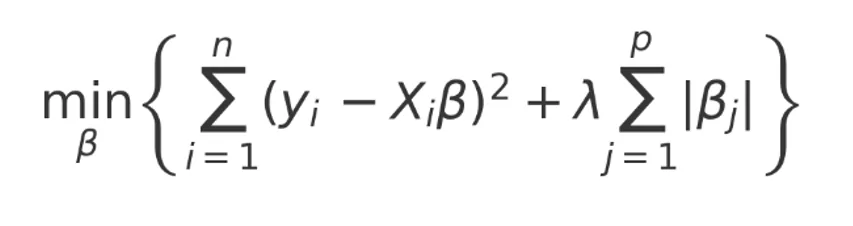



Ridge addressed multicollinearity through L2 regularization, minimizing:

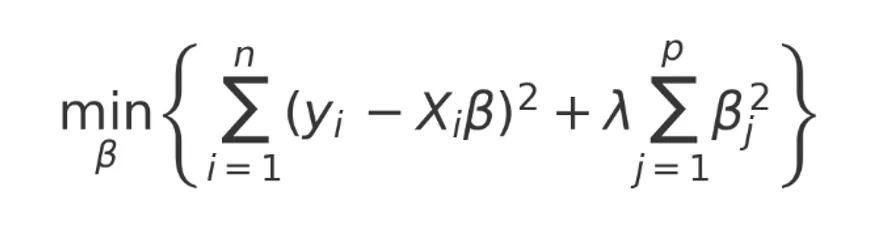



k-NN predicted outcomes based on proximity in feature space. Decision trees provided interpretable, hierarchical structures. However, because single decision trees are prone to overfitting, they were complemented by RF, an ensemble method aggregating multiple trees to counteract this limitation. GBM sequentially minimized residual errors to capture complex nonlinear relationships.

### Data Preprocessing

Categorical independent variables at baseline were summarized as absolute counts and percentages. Continuous baseline independent variables were reported as mean (SD) and assessed for extreme outliers; however, no winsorization was applied to preserve the original data distribution. Differences in WHO-FC between baseline and 12-month follow-up were assessed using a 2-sided paired *t* test. A 2-sided *P* value <.01 was considered statistically significant. The significance of Pearson correlation coefficients was assessed using the t-distribution for associations between WHO-FC at 12-month follow-up (the dependent variable) and each independent variable, as well as among all independent variables. Missing values in numeric variables were imputed using the median of each respective variable. Missing data frequencies for selected predictor variables and the primary outcome variable prior to imputation are reported in Multimedia Appendix 2. Median imputation was selected because of its robustness to outliers and its ability to preserve the complete dataset in this proof-of-concept analysis. Median imputation was performed before the dataset was split into training and test sets.

The primary end point is an ordinal outcome ranging from Class I to Class IV. For the purpose of this proof-of-concept study, WHO-FC was modeled using regression-based algorithms, treating class transitions as approximately equidistant to enable continuous outcome prediction. This approach treats WHO-FC as a quasi-continuous variable despite its ordinal nature. Considering the exploratory proof-of-concept design, the primary objective of modeling longitudinal changes in functional status, and the modest sample size with limited distribution across WHO-FC categories, a regression framework was considered a pragmatic modeling approach.

The dataset was randomly split into a training set (144/181, 79.6%) and an independent test set (37/181, 20.4%). Model training and hyperparameter tuning were conducted in R using grid search and 5-fold cross-validation. Within the training set, 5-fold cross-validation was performed by partitioning the data into 5 subsets, iteratively training on 4 folds and validating on the remaining one. The average performance across all folds was used to optimize hyperparameters and reduce the risk of overfitting. Given the modest sample size, no additional hold-out validation subset was created. Five-fold cross-validation within the training set served as the internal validation strategy, while the independent test set was used for final model evaluation and comparison of model performance.

### Model Evaluation

Model performance was assessed using 5 predefined metrics. Pearson correlation coefficient (*r*) was used to quantify the strength of the linear association between observed and predicted values, whereas the coefficient of determination (*R*^2^) quantified the proportion of outcome variance explained by the model. In addition, root mean squared error (RMSE), mean absolute error (MAE), and median absolute error (MedAE) were calculated to assess prediction error and overall model performance. RMSE reflects the average magnitude of prediction errors while placing greater weight on larger deviations. MAE represents the average absolute difference between observed and predicted values and is less sensitive to outliers than RMSE. MedAE summarizes the median absolute prediction error and provides a robust measure of typical prediction accuracy [38].

The respective formulas are:

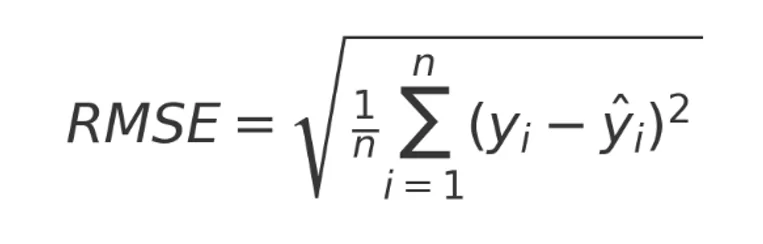

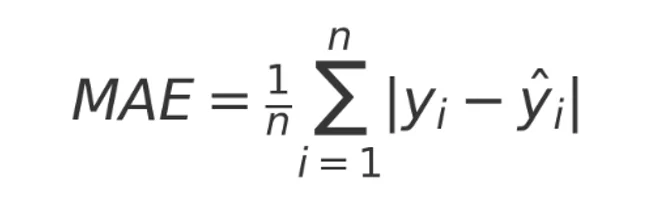

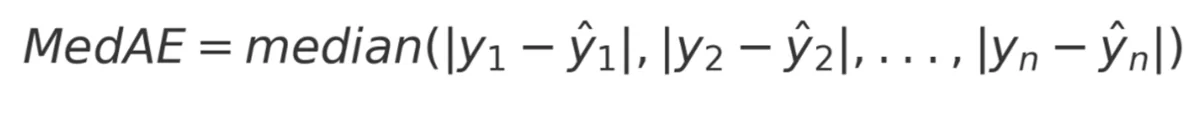







where yi and ŷi represent the observed and predicted relative changes in WHO-FC, respectively.

Final model performance was evaluated on the held-out test set following model development and tuning. Model selection was based on optimizing these predefined performance metrics. Specifically, the final model was chosen to maximize *r* and *R*^2^ while minimizing RMSE, MAE, and MedAE to balance predictive strength with error minimization.

The full modeling pipeline, including data preparation, model training, validation, and evaluation, is summarized schematically in Figure 1.

**Figure 1:**
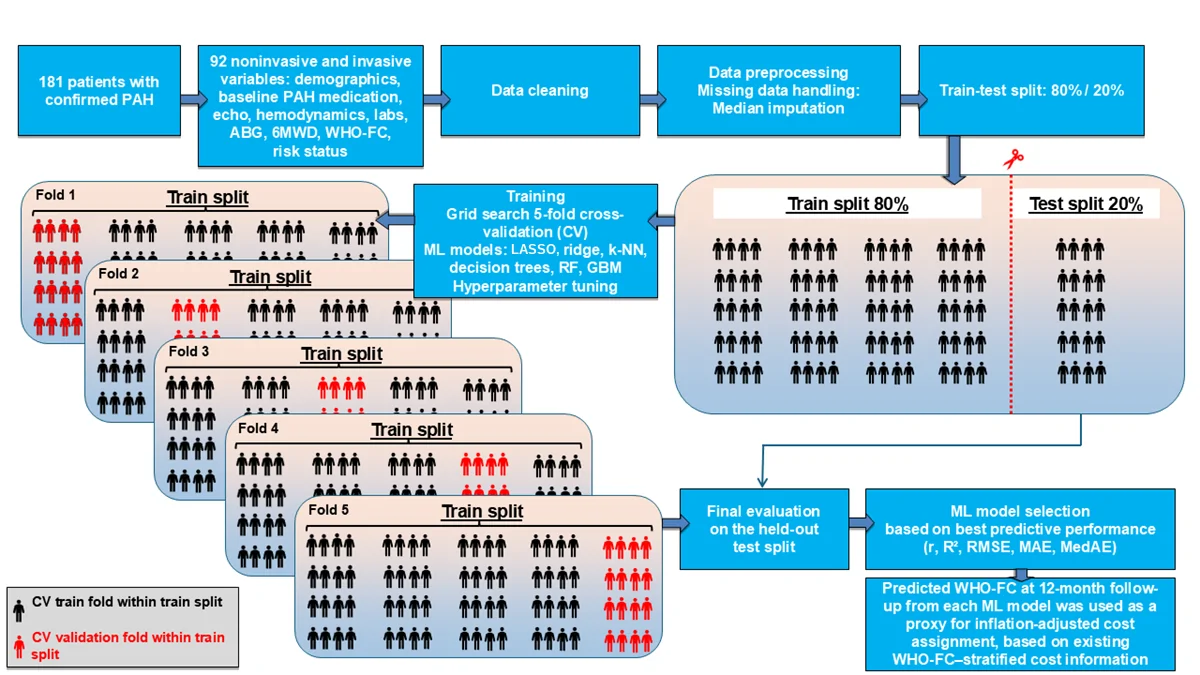
Schematic representation of the modeling pipeline. Data from 181 patients with pulmonary arterial hypertension and 92 baseline variables were split into training (144/181, 79.6%) and test (37/181, 20.4%) sets. Six machine learning algorithms were optimized using 5-fold cross-validation, followed by evaluation on the independent test set. Predicted World Health Organization functional class at 12-month follow-up was subsequently linked to stratified, inflation-adjusted health care cost categories. 6MWD: 6-minute walk distance; ABG: arterial blood gas; GBM: gradient boosting machine; k-NN: k-nearest neighbor; LASSO: Least Absolute Shrinkage and Selection Operator; MAE: mean absolute error; MedAE: median absolute error; ML: machine learning; PAH: pulmonary arterial hypertension; R^2^: coefficient of determination; RF: random forest; ridge: ridge regression; RMSE: root mean squared error; WHO-FC: World Health Organization functional class.

### WHO-FC–Based Health Care Cost Estimation

Each predicted WHO-FC value was deterministically linked to the corresponding inflation-adjusted total healthcare cost for the respective WHO-FC class. Cost mapping was formalized as:

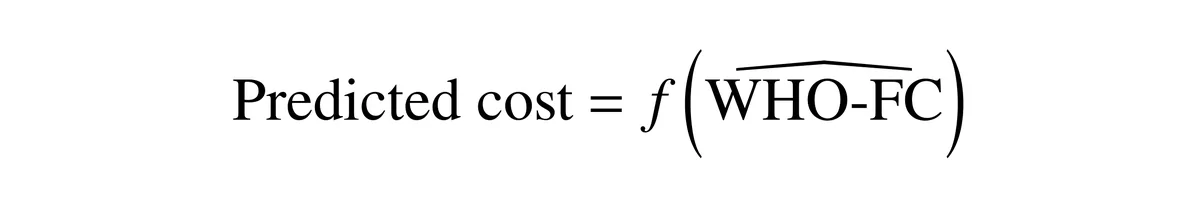



where f(·) denotes the deterministic mapping from the predicted WHO-FC to the corresponding validated WHO-FC–specific cost stratum. Thus, predicted WHO-FC served as the basis for estimating patient-level health care costs through mapping to validated WHO-FC–specific cost strata.

### Ethical Considerations

The study protocol was approved by the Ethics Committee of the Faculty of Medicine, University of Cologne, Germany (reference number 22-1318_2-retro). All patients provided written informed consent for the use and analysis of their data for research purposes. Patient privacy and confidentiality were maintained throughout the study. All data used for this analysis were deidentified, and no information that could identify individual participants is reported in this paper. Participants received no compensation for participation in this study. The study was conducted in accordance with the principles of the Declaration of Helsinki. The study is reported in accordance with the Transparent Reporting of a Multivariable Prediction Model for Individual Prognosis or Diagnosis (TRIPOD) statement. Given the sample size and feature dimensionality, this study is intended as a proof-of-concept and hypothesis-generating analysis.

## Results

### Baseline Patient Characteristics

A total of 181 patients with invasively confirmed PAH were included in the final analysis. The mean age was 62.5 (SD 16.5) years, and 64.6% (117/181) were female. The majority were diagnosed with idiopathic PAH (123/181, 68.0%), followed by PAH associated with connective tissue disease (40/181, 22.1%). Functional capacity was impaired at baseline, as reflected by a mean WHO-FC of 2.9 (SD 0.4) and a 6MWD of 340.2 (SD 106.9) meters. NT-proBNP levels were markedly elevated (mean 2559, SD 5196 ng/L), indicating advanced right ventricular strain. Hemodynamic measurements obtained by RHC confirmed severe pulmonary vascular disease, with mPAP of 46.0 (SD 14.0) mm Hg, PAWP of 10.5 (SD 3.9) mm Hg, PVR of 9.9 (SD 6.8) WU, and CI of 2.1 (SD 0.5) L/min/m^2^. Detailed baseline characteristics are presented in Table 2.

**Table 2:** Baseline characteristics of patients with newly diagnosed pulmonary arterial hypertension (N=181), including demographic, clinical, hemodynamic, laboratory, and echocardiographic parameters.

Demographics			Values	
Age (years), mean (SD)			62.5 (16.5)	
Sex, n (%)				
	Female	117 (64.6)		
	Male	64 (35.4)		
Weight (kg), mean (SD)			78.4 (18.8)	
Height (cm), mean (SD)			168.4 (8.8)	
BMI (kg/m^2^), mean (SD)			27.6 (6.3)	
BSA^a^ (m^2^), mean (SD)			1.9 (0.2)	
PAH^b^ subtype, n (%)				
	Idiopathic	123 (68.0)		
	Heritable	8 (4.4)		
	Drug-associated	4 (2.2)		
	Connective tissue disease	40 (22.1)		
	Congenital heart disease	3 (1.6)		
	HIV	2 (1.1)		
	Other	1 (0.6)		
Comorbidities and cardiovascular risk factors, n (%)				
	Hypertension	119 (65.7)		
	Diabetes	41 (22.7)		
	Coronary artery disease	51 (28.2)		
	BMI >30 kg/m^2^	53 (29.3)		
	Dyslipidemia	53 (29.3)		
	History of atrial fibrillation	41 (22.7)		
Functional status, mean (SD)				
	WHO-FC^c^	2.9 (0.4)		
	6MWD^d^ (m)	340.2 (106.9)		
Hemodynamic parameters, mean (SD)				
	Systolic PAP^e^ (mm Hg)	73.0 (21.7)		
	Diastolic PAP (mm Hg)	27.2 (10.3)		
	Mean PAP (mm Hg)	46.0 (14.0)		
	PAWP^f^ (mm Hg)	10.5 (3.9)		
	RAP^g^ (mm Hg)	7.9 (3.8)		
	CI^h^ (L/min/m^2^)	2.1 (0.5)		
	TPG^i^ (mm Hg)	35.3 (14.4)		
	DPG^j^ (mm Hg)	17.2 (11.0)		
	SVI^k^ (mL/m^2^/beat)	27.8 (8.5)		
	PVR^l^ (WU^m^)	9.9 (6.8)		
	PAC^n^ (mL/mm Hg)	1.2 (0.6)		
	SvO_2_^o^ (%)	62.3 (7.7)		
Laboratory parameters, mean (SD)				
	NT-proBNP^p^ (ng/L)	2559 (5196)		
	Creatinine (mg/dL)	1.09 (0.41)		
Echocardiographic parameters, mean (SD)				
	RA^q^ area (cm^2^)	23.9 (7.8)		
	RVEDD^r^ (mm)	43.5 (7.2)		
	PASP^s^ (mm Hg)	76.5 (21.4)		
	TAPSE^t^ (mm)	17.7 (4.4)		
	TAPSE/PASP (mm/mm Hg)	0.26 (0.12)		
	LVEDD^u^ (mm)	42.0 (6.3)		
	LVEF^v^ (%)	63.3 (6.4)		

^a^BSA: body surface area.

^b^PAH: pulmonary arterial hypertension.

^c^WHO-FC: World Health Organization functional class.

^d^6MWD: 6-minute walk distance.

^e^PAP: pulmonary arterial pressure.

^f^PAWP: pulmonary arterial wedge pressure.

^g^RAP: right atrial pressure.

^h^CI: Cardiac index.

^i^TPG: transpulmonary gradient.

^j^DPG: diastolic pulmonary gradient.

^k^SVI: stroke volume index.

^l^PVR: pulmonary vascular resistance.

^m^WU: Wood unit.

^n^PAC: pulmonary artery compliance.

^o^SvO₂: mixed venous oxygen saturation.

^p^NT-proBNP: N-terminal pro-B-type natriuretic peptide.

^q^RA: right atrial.

^r^RVEDD: right ventricular end-diastolic diameter.

^s^PASP: pulmonary artery systolic pressure.

^t^TAPSE: tricuspid annular plane systolic excursion.

^u^LVEDD: left ventricular end-diastolic diameter.

^v^LVEF: left ventricular ejection fraction.

### Change in WHO-FC

At 12-month follow-up, mean WHO-FC decreased significantly from 2.9 (SD 0.4) at baseline to 2.3 (SD 0.5; *P*<.01), corresponding to a shift from more advanced functional limitation (Class III) toward moderate impairment (Class II). The change was observed under standard-of-care therapy in accordance with the applicable guidelines at the time of patient presentation, with median imputation applied for missing WHO-FC values.

### Bivariate Correlation of Baseline Variables With WHO-FC

To explore the relationship between baseline parameters and the target variable, bivariate Pearson correlation coefficients were calculated for all 63 metric input variables. The complete correlation matrix and corresponding significance matrix are provided in Multimedia Appendix 3. Among the variables, baseline WHO-FC showed the strongest correlation with WHO-FC at follow-up (*r*=+0.40; *P*<.01). 6MWD was weakly but significantly correlated (*r*=–0.22; *P*<.01), whereas NT-proBNP showed no significant correlation (*r*=–0.08; *P*=.29). Additional correlations among baseline variables suggested multicollinearity. No variables were excluded or aggregated, as multicollinearity was addressed through regularization techniques within the ML models.

### Model Performance in Predicting Relative Change in WHO-FC

Six supervised ML algorithms were trained to predict the relative change in WHO-FC from baseline to 12-month follow-up, using exclusively baseline variables as input. Predicted change rates were subsequently converted to predicted WHO-FC values at 12-month follow-up. A summary of performance metrics is provided in Table 3.

**Table 3:** Predictive performance of machine learning models for relative change in World Health Organization functional class from baseline to the 12-month follow-up. Reported metrics include r, R^2^, root mean squared error, mean absolute error, and MedAE.

Model	*r* ^a^	*R* ^2^ ^b^	RMSE^c^	MAE^d^	MedAE^e^
LASSO^f^ regression	0.301	0.091	0.130	0.114	0.117
Ridge regression	0.259	0.067	0.133	0.117	0.109
k-nearest neighbors	0.401	0.161	0.125	0.106	0.121
Decision trees	0.293	0.086	0.139	0.112	0.120
Random forest	0.299	0.089	0.133	0.114	0.113
Gradient boosting	0.288	0.083	0.134	0.120	0.140

^a^*r*: Pearson correlation coefficient.

^b^*R*^2^: coefficient of determination.

^c^RMSE: root mean squared error.

^d^MAE: mean absolute error.

^e^MedAE: median absolute error.

^f^LASSO: Least Absolute Shrinkage and Selection Operator regression.

Following evaluation on the independent test set, k-NN showed the highest performance among the evaluated models, with *r*=0.401 and *R*^2^=0.161. Prediction errors were low, with RMSE=0.125 and MAE=0.106. LASSO produced the second-best results (*r*=0.301; *R*^2^=0.091; RMSE=0.130; MAE=0.114). Ensemble models, including RF and GBM, as well as single decision trees, showed comparable predictive strength (*r*=0.299, 0.288, and 0.293, respectively), while ridge yielded the lowest performance (*r*=0.259). Across all models, RMSE and MAE exhibited limited variation, indicating stable model behavior. However, absolute predictive performance, as measured by *r* and *R*^2^, differed moderately between models.

To further interpret the second best-performing model, the penalized effect sizes derived from LASSO are visualized in Figure 2 [34,36]. LASSO identified a sparse set of 8 predictors with nonzero coefficients, while all remaining variables were reduced to zero by L1 regularization.

**Figure 2:**
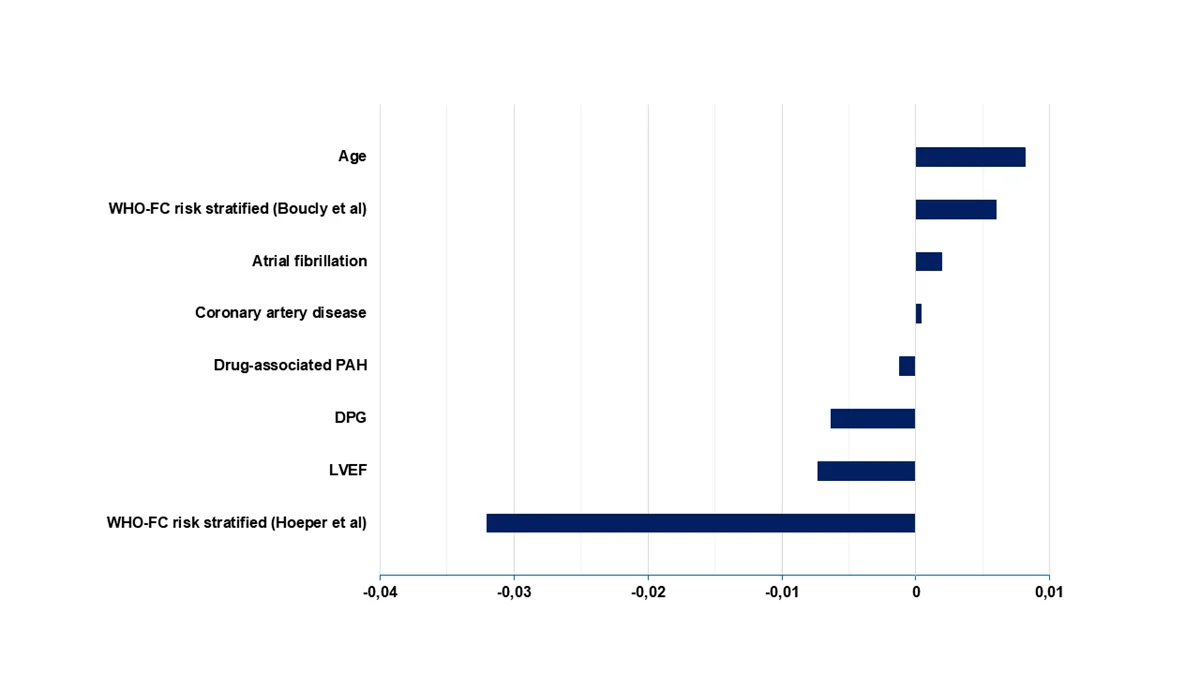
Effect sizes represent the penalized regression coefficients of the final Least Absolute Shrinkage and Selection Operator model, indicating the direction and relative strength of association with the predicted relative change in World Health Organization functional class from baseline to 12-month follow-up. Shown are the 8 variables retained by L1 regularization. Positive coefficients indicate a greater predicted relative increase in World Health Organization functional class (worsening functional status), whereas negative coefficients indicate a greater predicted relative decrease (improvement in functional status). The WHO-FC risk stratification variables were based on the approaches described by Hoeper et al [34] and Boucly et al [36]. DPG: diastolic pulmonary gradient; LVEF: left ventricular ejection fraction; PAH: pulmonary arterial hypertension; WHO-FC: World Health Organization functional class.

Among the selected nonbinary predictors, positive effect sizes were observed for age (+0.008). Negative effect sizes were found for WHO-FC at baseline (–0.032), stratified using the refined four-strata model [34,35], left ventricular ejection fraction (LVEF; –0.007), and diastolic pulmonary gradient (DPG; –0.006).

### Predicted WHO-FC–Based Cost Estimation

To translate model predictions into health care cost estimates, each predicted WHO-FC value was mapped to validated cost categories stratified by functional class (I-IV), based on inflation-adjusted data from Dufour et al [11]. Predicted mean pharmacy and medical costs were summed to derive total estimated health care costs PPPM.

Table 4 presents the predicted costs at 12-month follow-up across all ML models.

**Table 4:** Predicted health care costs based on World Health Organization functional class proxies at 12-month follow-up across machine learning models. Shown are mean pharmacy, medical, and total costs per patient per month, with total costs representing the sum of pharmacy and medical costs. Values are inflation-adjusted to 2024 US dollars using the Medical Care Consumer Price Index.

Cost category	LASSO^a^ regression (US $)	Ridge regression (US $)	k-nearest neighbors (US $)	Decision trees (US $)	Random forest (US $)	Gradient boosting (US $)
Pharmacy	5865	5884	5903	5859	5884	5872
Medical	6730	6654	6578	6755	6654	6704
Total	12,595	12,538	12,481	12,614	12,538	12,576

^a^LASSO: Least Absolute Shrinkage and Selection Operator regression.

k-NN, which achieved the highest predictive performance, forecasted mean pharmacy costs of US $5903, medical costs of US $6578, and total costs of US $12,481 PPPM. Cost estimates were comparable across models, with mean total costs ranging from US $12,481 predicted by k-NN to US $12,614 predicted by decision trees, indicating relatively consistent model behavior in cost estimation. k-NN produced the cost prediction closest to the reference value of US $12,332 based on observed WHO-FC at 12-month follow-up, while decision trees deviated the most, consistent with their lower predictive performance.

Beyond model-based predictions, total inflation-adjusted PPPM health care costs based on observed WHO-FC at follow-up (US $12,332) remained nearly unchanged compared with baseline (US $12,329), despite a significant improvement in mean WHO-FC from 2.9 (SD 0.4) to 2.3 (SD 0.5; *P*<.01). While mean PPPM pharmacy costs decreased from US $6018 at baseline to US $5852 at 12-month follow-up, mean PPPM medical costs increased slightly from US $6311 to US $6479. Totals may differ slightly from the sum of the displayed components because of rounding. Figure 3A-C shows the calculated aggregate monthly pharmacy, medical, and total health care costs at baseline and 12-month follow-up for each WHO-FC stratum (I-IV) and for the overall cohort, inflation-adjusted to 2024 US dollars. Figure 3D shows the number of patients in each WHO-FC stratum at both time points.

**Figure 3:**
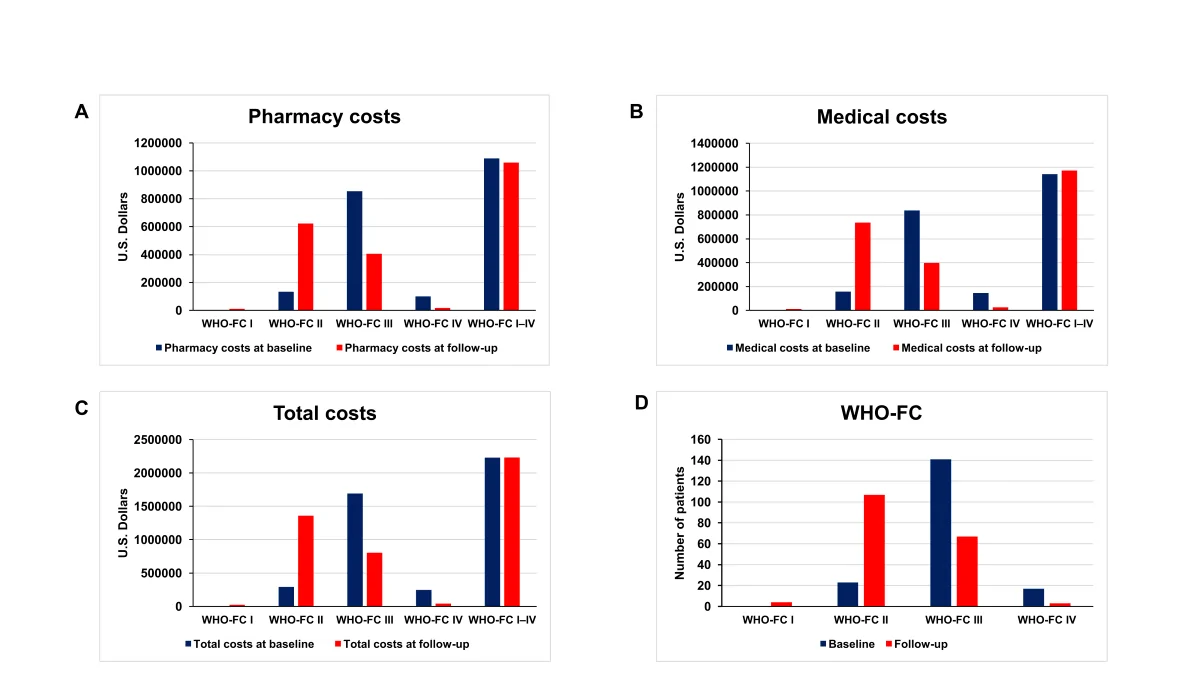
Calculated aggregate monthly (A) pharmacy, (B) medical, and (C) total health care costs at baseline and 12-month follow-up, shown for each WHO-FC stratum (I–IV) and for the overall cohort. (D) Number of patients in each WHO-FC stratum at both time points. For each cost category, stratum-specific costs were calculated by multiplying the number of patients in that stratum at the corresponding time point by the published WHO-FC–specific mean per-patient-per-month costs reported by Dufour et al [11]. Overall cohort costs were calculated by summing across strata. All costs are expressed in US dollars and inflation-adjusted from 2014 to 2024 values using the Medical Care Consumer Price Index. Blue bars indicate baseline; red bars indicate 12-month follow-up. WHO-FC: World Health Organization functional class.

## Discussion

### Principal Findings

This pilot study evaluated an ML-based framework linking prediction of 12-month WHO-FC with health care cost estimation in PAH, a disease associated with substantial clinical and economic burden [1,5,14,16,39-41]. Using routinely collected baseline data, the models showed moderate predictive performance and generated broadly comparable WHO-FC–based cost estimates across algorithms. These findings suggest that clinical prediction and economic modeling can be combined using WHO-FC, a clinically established marker of disease severity and health care costs [1,9,11,34-36]. However, given the moderate predictive performance observed in this proof-of-concept study, further validation is required before clinical or health-system implementation.

### ML-Based Prediction and Cost Anticipation

Our results suggest that ML models can provide a preliminary estimate of future WHO-FC and associated cost strata from baseline clinical data. However, the modest explained variance indicates that the current framework should not be interpreted as supporting individual-level clinical or economic decision-making. Rather, the findings provide proof-of-concept evidence that clinical prediction models may be linked to downstream cost estimates in PAH. Previous ML studies in PH and PAH have predominantly focused on diagnostic classification or risk stratification using binary or categorical end points, often reporting high discriminative performance [42-45]. In contrast, our models achieved moderate predictive performance while predicting longitudinal changes in WHO-FC over a 12-month follow-up from baseline clinical data. Direct comparison of predictive performance should therefore be interpreted with caution because of differences in prediction targets, underlying data, outcome complexity, and evaluation metrics. Recent ML studies on health care cost prediction have largely been conducted in broader health care settings using routinely collected clinical and administrative data [46,47]. To our knowledge, no previous study has combined ML-based prediction of future WHO-FC with downstream health care cost estimation in PAH.

### Functional Improvement and Health Care Cost Patterns

Despite significant improvement in mean WHO-FC from 2.9 to 2.3, estimated total PPPM costs remained nearly unchanged. This finding likely reflects the relatively flat cost gradient between WHO-FC II and III in the reference dataset by Dufour et al [11], where lower pharmacy costs in WHO-FC II are offset by higher medical costs. Thus, functional improvement within adjacent WHO-FC strata may not necessarily translate into proportional short-term reductions in total direct health care costs. Prior studies suggest that cost gradients are more pronounced at the extremes of functional status, particularly between WHO-FC IV and III and between WHO-FC II and I [9,11]. Therefore, WHO-FC–based cost estimation may be most informative when identifying patients at risk of remaining in or progressing to higher cost strata. However, because health care expenditures are influenced by treatment intensity, hospitalizations, comorbidities, and indirect costs, WHO-FC alone cannot fully capture individual cost variability [9,11,16].

### Cost Profile Stratification as a Complement to Risk Stratification

WHO-FC may serve as a practical bridge between clinical risk stratification and health economic modeling because it is a central component of contemporary PAH risk assessment, is prognostically relevant, easily assessable in routine clinical practice without specialized diagnostic equipment, internationally standardized, and consistently associated with health care costs and HCRU across multiple studies and health care settings [1,9,11,16,21,22,34-36,48,49]. Consequently, WHO-FC–based cost profiling should be viewed as complementary to established PAH risk stratification by extending clinical assessment with a health economic perspective.

However, although WHO-FC is an established surrogate of disease severity and has consistently been associated with health care costs in PAH, it does not fully capture the variability of health care expenditures among patients within the same functional class. Health care costs are influenced by disease severity but are not exclusively determined by it. Accordingly, the proposed framework should be interpreted as providing patient-level health care cost estimates through mapping predicted WHO-FC to established WHO-FC–specific cost strata rather than directly predicting individual health care expenditures.

### Methodological Approach and Interpretability

Several methodological features of our approach enhance its interpretability and potential for clinical adoption. First, the inclusion of 6 different ML models with cross-validation and independent test evaluation strengthens internal validity [50,51]. Second, the LASSO-based feature selection approach offers inherent model transparency by identifying features with nonzero coefficients, thereby highlighting variables of predictive relevance and potential clinical interest [51-54]. This enhances trust in the model and facilitates communication with clinicians, which is a key factor for eventual integration into electronic health record (EHR) systems or clinical decision support tools.

Although k-NN achieved the highest predictive performance in this study, its clinical interpretability is inherently limited. Unlike coefficient-based models such as LASSO regression, k-NN generates predictions based on similarity relationships between patients in a multidimensional feature space and does not provide explicit model coefficients, effect estimates, or readily interpretable predictor contributions. Consequently, the factors driving individual predictions cannot be directly quantified. This trade-off between predictive performance and interpretability represents an important consideration for future clinical implementation, where transparent decision-support tools may be preferred.

Importantly, treating WHO-FC as an approximately continuous outcome reflects a methodological trade-off. While this approach facilitated regression-based modeling of longitudinal changes in functional status, it does so at the expense of some ordinal information and interpretive granularity [55]. Alternative ordinal or probabilistic modeling approaches may provide closer methodological alignment with the underlying outcome structure and should therefore be evaluated in future studies.

### Transferability, Generalizability, and System-Specific Implications

The use of claims-derived cost data represents an important limitation. Although costs were inflation-adjusted and expressed in 2024 US dollars, absolute estimates are unlikely to be directly transferable to European or other non-US health care systems because reimbursement structures, drug pricing, hospitalization costs, and access pathways differ substantially [11,56-60]. Therefore, the current cost estimates should be interpreted as illustrating the framework rather than providing system-specific budget impact estimates.

Future adaptations should incorporate country-specific cost sources, such as German statutory health insurance data, diagnosis-related group (DRG) tariffs, or payer-specific claims datasets. In practice, the underlying ML model could remain unchanged, while the cost-mapping component would be recalibrated using health care system–specific cost data and reimbursement structures. Inclusion of indirect costs, including absenteeism, disability, and informal caregiving, would further improve the relevance of the framework for societal-perspective analyses [9,16].

### Strengths and Limitations

Strengths of this study include the hemodynamic confirmation of PAH diagnosis and the comprehensive collection of noninvasive baseline variables without clinical preselection. A clearly defined preprocessing strategy was applied, and missing data were imputed using median substitution to preserve the full dataset. Crucially, model performance was evaluated on an independent test set following cross-validation and hyperparameter tuning, helping to assess generalizability and reduce the risk of overfitting. As a pilot study, this work represents an early application of ML for estimating future health care costs in PAH based on routinely collected clinical data. These findings should be interpreted as preliminary and hypothesis-generating, particularly in light of the modest predictive performance and single-center design.

Limitations include the retrospective, single-center design, the absence of external validation in an independent multicenter cohort from other clinical centers, and the modest sample size, all of which may affect the generalizability of the results, as well as the relatively high feature-to-sample ratio, which may increase the risk of overfitting despite internal validation. Patients with borderline hemodynamic profiles, who would now qualify as PAH according to the updated 2022 ESC and ERS guidelines, were not included, which limits applicability to milder disease stages. Predicted WHO-FC values were linked to cross-sectional health care cost estimates from a US claims database, which may reduce transferability to European health care systems despite inflation adjustment. Additionally, the use of WHO-FC as a proxy for cost estimation may not fully capture individual variability in health care expenditures. Because WHO-FC was modeled as a quasi-continuous outcome despite its ordinal structure, additional uncertainty may have been introduced into both the prediction process and the subsequent assignment of predicted outcomes to cost strata. Finally, the limited interpretability of the best-performing k-NN model may constrain clinical transparency and implementation.

### Future Directions

Building on this proof of concept, future studies should validate the approach in larger multicenter cohorts using region-specific cost data to improve generalizability. Future models integrating WHO-FC with additional objective clinical markers, such as NT-proBNP or hemodynamic parameters, as well as socioeconomic characteristics, indirect costs, and longitudinal follow-up data, may further improve the precision of health care cost estimation, predictive accuracy, and overall health economic relevance. For practical implementation, ML-based cost prediction tools should be embedded into EHR systems or health care management platforms via dedicated technical interfaces. Once externally validated, such tools may support prospective economic planning aligned with clinical risk stratification and promote resource-efficient and individualized care in PAH. Future studies may also benefit from combining predictive accuracy with explainable ML approaches to improve model transparency and facilitate clinical implementation.

### Conclusions

This study demonstrates the feasibility of integrating ML-based prediction with health care cost estimation in PAH. Using routinely collected baseline clinical data, the proposed framework provides a proof-of-concept for early approximation of functional status and associated health care expenditures. While predictive performance was moderate, the findings suggest that ML-based clinical prediction may be combined with downstream health care cost estimation. Given the pilot nature of this study, prospective validation in larger, multicenter cohorts is required before clinical or health-system implementation. Successful integration into real-world health care systems and EHR platforms will require standardized data availability and technical interoperability.

## Data Availability

The data underlying this study are available from the corresponding author upon reasonable request, subject to ethical and privacy considerations.

## Appendix

Multimedia Appendix 1 Inflation adjustment of health care costs based on World Health Organization functional class. Health care costs based on World Health Organization functional class, originally reported in 2014 US dollars, were adjusted to 2024 values using the Medical Care Consumer Price Index, applying an adjustment factor of 1.291. Pharmacy, medical, and total per patient per month costs are shown separately for each World Health Organization functional class. Consumer Price Index values were obtained from official US inflation statistics for the years 2014 and 2024. Totals may differ slightly from the sum of the displayed components because of rounding.

Multimedia Appendix 2 Missing data frequencies for selected predictor variables and the primary outcome variable. Missing data frequencies prior to median imputation. Values are reported as the number and percentage of missing observations relative to the total study cohort (N=181).

Multimedia Appendix 3 Correlation heatmaps for baseline variables and World Health Organization functional class at 12-month follow-up. (A) Correlation heatmap showing the strength of association between baseline independent variables and World Health Organization functional class at 12-month follow-up. (B) Corresponding heatmap displaying *P* values for the same variable-target associations.

## Abbreviations

6MWD: six-minute walk distance
CI: cardiac index
CPI: consumer price index
DPG: diastolic pulmonary gradient
DRG: diagnosis-related group
EHR: electronic health record
ERS: European Respiratory Society
ESC: European Society of Cardiology
GBM: gradient boosting machine
HCRU: health care resource usage
k-NN: k-nearest neighbor
LASSO: Least Absolute Shrinkage and Selection Operator
LVEF: left ventricular ejection fraction
MAE: mean absolute error
MedAE: median absolute error
ML: machine learning
mPAP: mean pulmonary arterial pressure
NT-proBNP: N-terminal pro-B-type natriuretic peptide
PAH: pulmonary arterial hypertension
PASP: pulmonary artery systolic pressure
PAWP: pulmonary arterial wedge pressure
PH: pulmonary hypertension
PPPM: per patient per month
PVR: pulmonary vascular resistance
r: Pearson correlation coefficient
R^2^: coefficient of determination
RF: random forest
RHC: right heart catheterization
RMSE: root mean squared error
TAPSE: tricuspid annular plane systolic excursion
TRIPOD: Transparent Reporting of a Multivariable Prediction Model for Individual Prognosis or Diagnosis
WHO-FC: World Health Organization functional class
WU: Wood unit
