# Supplementary material for: A Machine Learning Framework for Predicting Functional Status and Estimating Health Care Costs in Pulmonary Arterial Hypertension: Retrospective Cohort Study
Source: JMIR Med Inform. 2026 Oct 15;14:e97372. doi: 10.2196/97372 (PMC13720409; doi:10.2196/97372)
Supplement: Supplementary file 1 [file medinform_v14i1e97372_app1.docx]

**Multimedia Appendix 1. Inflation adjustment of healthcare costs based on WHO-FC.**

Healthcare costs based on WHO-FC originally reported in 2014 U.S.$ were adjusted to 2024 values using the Medical Care CPI, applying an adjustment factor of 1.291. Pharmacy, medical, and total PPPM costs are shown separately for each WHO-FC. CPI values were obtained from official U.S. inflation statistics for the years 2014 and 2024.

| **WHO-FC** | **Cost Category** | **Original Costs (2014 U.S.$)** | **CPI Adjustment Factor (CPI 2024 / CPI 2014)** | **Adjusted Costs (2024 U.S.$)** |
| --- | --- | --- | --- | --- |
| **I** | Pharmacy | $2,301 | 1.291 | $2,971 |
| **I** | Medical | $2,401 | 1.291 | $3,100 |
| **I** | Total | $4,703 | 1.291 | $6,072 |
| **II** | Pharmacy | $4,514 | 1.291 | $5,828 |
| **II** | Medical | $5,331 | 1.291 | $6,882 |
| **II** | Total | $9,845 | 1.291 | $12,710 |
| **III** | Pharmacy | $4,696 | 1.291 | $6,063 |
| **III** | Medical | $4,604 | 1.291 | $5,944 |
| **III** | Total | $9,300 | 1.291 | $12,006 |
| **IV** | Pharmacy | $4,575 | 1.291 | $5,906 |
| **IV** | Medical | $6,655 | 1.291 | $8,592 |
| **IV** | Total | $11,230 | 1.291 | $14,498 |

**Abbreviations**

CPI, Consumer Price Index; PPPM, per-patient-per-month; WHO-FC, World Health Organization functional class.
