# Supplementary material for: A Machine Learning Framework for Predicting Functional Status and Estimating Health Care Costs in Pulmonary Arterial Hypertension: Retrospective Cohort Study
Source: JMIR Med Inform. 2026 Oct 15;14:e97372. doi: 10.2196/97372 (PMC13720409; doi:10.2196/97372)
Supplement: Supplementary file 2 [file medinform_v14i1e97372_app2.docx]

**Multimedia Appendix 2. Missing data frequencies for selected predictor variables and the primary outcome variable.**

Missing data frequencies prior to median imputation. Values are reported as the number and percentage of missing observations relative to the total study cohort (n=181).

| **Variable** | **Missing values, n (%)** |
| --- | --- |
| **Baseline demographics** | |
| Age | 0 (0.0%) |
| Gender | 0 (0.0%) |
| Weight | 0 (0.0%) |
| Height | 0 (0.0%) |
| BSA | 0 (0.0%) |
| BMI | 0 (0.0%) |
| **Baseline hemodynamics** | |
| PAPmean | 2 (1.1%) |
| PAWP | 3 (1.7%) |
| PVR | 5 (2.8%) |
| TPG | 5 (2.8%) |
| PAPsys | 8 (4.4%) |
| CO | 8 (4.4%) |
| PAPdias | 9 (5.0%) |
| DPG | 13 (7.2%) |
| CI | 15 (8.3%) |
| RAP | 26 (14.4%) |
| **Baseline functional and laboratory variables** | |
| WHO-FC at baseline | 0 (0.0%) |
| Creatinine | 5 (2.8%) |
| Urea | 10 (5.5%) |
| NT-proBNP | 11 (6.1%) |
| CRP | 12 (6.6%) |
| INR | 13 (7.2%) |
| 6MWD | 26 (14.4%) |
| **Baseline echocardiographic variables** | |
| TAPSE | 9 (5.0%) |
| PASP | 10 (5.5%) |
| LV-EF | 10 (5.5%) |
| RA area | 12 (6.6%) |
| TAPSE/PASP | 12 (6.6%) |
| LVEDD | 15 (8.3%) |
| RVEDD | 15 (8.3%) |
| **Primary outcome variable at 12-month follow-up** | |
| WHO-FC at 12-month follow-up | 8 (4.4%) |

**Abbreviations**

6MWD, 6-minute walk distance; BMI, body mass index; BSA, body surface area; CI, cardiac index; CO, cardiac output; CRP, C-reactive protein; DPG, diastolic pulmonary gradient; INR, international normalized ratio; LV-EF, left ventricular ejection fraction; LVEDD, left ventricular end-diastolic diameter; NT-proBNP, N-terminal pro-brain natriuretic peptide; PAPdias, diastolic pulmonary arterial pressure; PAPmean, mean pulmonary arterial pressure; PAPsys, systolic pulmonary arterial pressure; PASP, pulmonary artery systolic pressure; PAWP, pulmonary artery wedge pressure; PVR, pulmonary vascular resistance; RA area, right atrial area; RAP, right atrial pressure; RVEDD, right ventricular end-diastolic diameter; TAPSE, tricuspid annular plane systolic excursion; TPG, transpulmonary gradient; WHO-FC, World Health Organization functional class.
